# Studying the aging of Laponite suspensions using extensional rheology

**DOI:** 10.1140/epje/s10189-022-00244-9

**Published:** 2022-11-16

**Authors:** M. J. Hayes, M. I. Smith

**Affiliations:** School of Physics, University of Nottingham, University Park, Nottingham, NG7 2RD UK

## Abstract

The effect of aging on the break-up dynamics of Laponite suspensions was studied in an extensional geometry. It was found that samples of increased age undergo stronger necking at the midpoint. The thinning of samples, driven purely by motion of the plates, was compared with standard shear rheology to understand how the dynamics are related to the sample properties. The Laponite suspensions exhibit a growing stress overshoot with monotonically decreasing yield strain as they age. However, it is shown that the thinning curves in extension are only a good indicator of the sample’s static yield stress, being insensitive to its yield strain. These measurements suggest that following an initial linear visco-elastic regime, samples accumulate significant plastic deformations prior to the complete yielding of the sample. The implications of this for the importance of assessing changes to the ductile—brittle nature of samples are also discussed.

**Graphical abstract:**

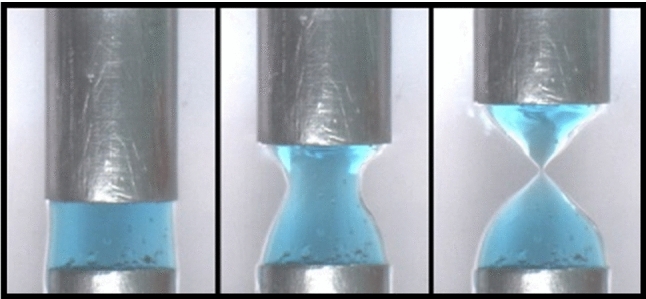

**Supplementary Information:**

The online version contains supplementary material available at 10.1140/epje/s10189-022-00244-9

## Introduction

Industrial processing of yield stress fluids frequently results in the break-up or separation of two masses of a fluid, e.g., the detachment of a droplet during a dispensing application or two surfaces coated with a liquid being pulled apart. Whilst measurements using a shear rheometer can extract precise rheological properties, other simpler tests are often preferred by industry to characterise or provide quality control of samples [1].

One approach makes use of the detachment of a pendant droplet from a needle. Measuring the weight of the detached droplet enables an estimate of the applied forces at the neck of the droplet, which when combined with visual measurements of the thinning, allows certain rheological properties, such as the yield stress to be extracted [2, 3]. The size of drop and hence applied stress can also be modified to some degree by altering the imposed flowrate [3]. The strain rate, however, varies throughout the experiment, controlled by gravity and the rheological properties.

Another approach is to use an extensional rheometer [4] which consists of two cylindrical plates, with a layer of liquid sandwiched between. As the plates are separated, the thinning and shape of the resulting break-up behaviour are monitored with either a laser micrometre or a camera to monitor the changes in sample profile. Often as the fluid is deformed, a stable bridge will develop between two liquid masses that wet each of the plates. This bridge then thins under the action of capillary pressure enabling the applied stress to be calculated from the bridge radius [5, 6].

However, yield stress fluids may sometimes break-up long before the formation of such a bridge. Sometimes the yield stress of the fluid is of sufficient magnitude that surface tension induced thinning is negligible or at least confined to a very small period and region of the flow just before break-up. Recently, Zhang et al. [7] were able to perform an extensional measurement, using a shear rheometer with a sufficiently large plate that they could directly measure the normal forces. To achieve this, they had to use boundary conditions that created almost complete slip at the boundaries. Their measurements showed that the shear and extensional yield stress, whilst always proportional, were related by different constants depending on the type of fluid studied.

Even when no measurement of the normal stress is possible, important information about the sample properties can still sometimes be deduced from observation of the sample’s break-up behaviour using extensional rheometry. For example, in several complex fluids, visual observation of fluids subjected to an extensional deformation has allowed researchers to assess changes in brittleness/ductility [8–10]. Recent theoretical works have also suggested that as samples age, yielding in amorphous materials results in a change between ductile and brittle behaviour [11, 12]. These studies predict that the brittleness of a sample is related to a stress–strain curve increasingly defined by a stress overshoot measured under homogeneous shear [12]. Therefore, quantifying these changes in a simple way could provide an indication of the age of a sample.

In this study, we focus on how the aging of a Laponite suspension affects the observed break-up behaviour in extension. Laponite is a synthetic clay that is commonly used as a rheology modifier [13]. It consists of disc-like particles of thickness  ~ 1 nm and radius  ~ 25 nm [14]. In suspension, these particles develop a negative face charge and smaller charge on the rim which is positive at high pH. The magnitude and sign of this charge can vary with temperature, pH and Na^+^ concentration [15]. This asymmetry means particles can associate either in face to face or face to rim interactions [16]. Depending on the conditions (concentration, pH, electrolyte concentration), it can form a variety of different phases and states including a Wigner glass, a DHOC glass, an isotropic liquid, an empty liquid, an equilibrium gel and a nematic phase [17–19].

Laponite suspensions often exhibit a yield stress and are strongly thixotropic, with substantial changes in properties observed over the timescale of hours. However, studies have shown that some samples have properties that continue to evolve for months or years after preparation [17, 20]. When subjected to shear the samples undergo a process of rejuvenation in which the microstructure of the sample is altered, and the β-relaxation time or measured moduli decrease [21, 22]. Following this, in the absence of an external applied stress, the same sample’s rheological properties, increase again with waiting time, resulting in power law growth of the relaxation time [14, 23] and elastic modulus [24–26]. Strongly pre-shearing a non-ergodic fluid prior to an experimental measurement is a common approach to erase a sample’s history dependence [1]. However, in the case of Laponite, freshly prepared samples have been shown to age very differently to strongly rejuvenated ones [19, 27]. The exact rejuvenation protocol, including sample history prior to the protocol, must therefore be carefully considered for a meaningful comparison to be made between experiments.

## Materials and methods

### Sample preparation

Laponite RD (ms16, Conservation Resources) was dried in a vacuum oven at 150 °C for 16 h. Stock suspensions of Laponite were then prepared by dissolution in deionised water containing 10^–4^ M NaCl. The pH of the suspension was then adjusted to pH 10 by adding 0.1 M NaOH dropwise. Suspensions of 3, 3.5 and 4 wt% were prepared, and then sealed in large bottles and left undisturbed for a period  ~ 2 weeks prior to use. Just before each set of experiments 50 ml of suspension was removed and mixed with 40µL of blue food colouring and thoroughly mixed through mechanical agitation and sonication.

Shear and extension measurements were performed with a Malvern Kinexus Pro Shear Rheometer. To study the aging of the Laponite suspensions, samples had to be left undisturbed in the rheometer for up to  ~ 16 h. Immersing both the sample and rheometer plates in oil prevented the evaporative loss of water over such long timescales [24, 28].

For the extension measurements, the lower 10-mm-diameter plate of the rheometer was mounted inside a transparent square cross section Perspex tube (see supplementary information). The tube was sealed at the bottom and open at the top and had a small access port in one of the side walls which was covered with a rubber seal. The upper plate was then moved to an initial gap height H_0_ of 5 mm and the tube was filled with Silicone oil (density 0.963 gcm^−3^)—chosen to nearly match the density of the Laponite samples ( ~ 1.01gcm^−3^). The Laponite suspension was then loaded into a Luer-Lock syringe and, using an 8 cm long G21 hypodermic needle, injected through the rubber seal between the rheometer plates.

The preparation, mixing with food colouring, and injection of our sample results in a complex stress history. Angelini et al. [27] showed that strongly sheared samples can exhibit different aging behaviour to freshly prepared samples. The process of injecting the Laponite strongly shears the sample just prior to the experiment resulting in rejuvenation. We assume that the waiting time (*t*_*w*_) measured with reference to this step is the dominant control variable for an understanding of the subsequent aging behaviour of the sample. This decision is supported by the reproducibility of our data and apparent insensitivity to the exact time between mixing with food colouring and injection.

Injecting the sample, we overfill the gap between the plates. To trim the sample, the upper rheometer plate is equipped with a PTFE collar (see supplementary info) that can slide downwards over the sample and onto the bottom plate, removing any excess fluid. Whilst this method works well, it limits the range of possible concentrations to  ~ 3–4wt%. Too low and the sample does not trim well; too high and the sample contact line is not well pinned during the experiment and undergoes slip. As the higher concentration samples aged we also encountered issues with slip that prevented direct quantitative comparison. After an appropriate waiting time, the upper plate is then moved upwards with a constant Hencky strain rate $\left({\dot{H}}/H_{0}\right)$ of either 0.08 or 0.008 s^−1^, where H_0_ is the initial gap between plates and ${\dot{H}}$ is the vertical velocity of the upper plate. These rates were selected to be fast enough that minimal aging of the sample happens over the course of the experiment [29] but slow enough to avoid strong viscous dissipation [30].

Whilst the normal forces acting on the upper plate are too small to measure, the experiment is filmed with a camera (Point Grey, Flea3) so that the break-up behaviour can be analysed. Automated image analysis of the plate separation and diameter of the midpoint was performed using python and OpenCV by subtracting two colour channels from each other to highlight the appropriate feature (plates or fluid), thresholding the image and then measuring the width of the sample at different heights. The midpoint was located as the narrowest region of the fluid.

Shear measurements were performed using a conventional 50 mm cone and plate (cone angle 1°). To immerse the sample in oil, we designed a 60-mm-diameter removable cylindrical ring which could be reversibly attached to the bottom plate. For these measurements, the Laponite suspension was deposited on the bottom plate via the same syringe and needle as before. The upper plate was brought to the trim height and excess sample removed. No further pre-shear was applied, in order to as closely as possible match the conditions in the extensional experiment. The ring was attached and the gap around the rheometer plate filled with Dodecane oil—chosen for its low viscosity (1.34 mPas) [24, 28]. After the appropriate waiting time, the samples were then measured using oscillatory or continuous shear. Control experiments showed that the difference in stress measured with or without the Dodecane oil was not significant.

## Results and discussion

### Initial observations and of break-up behaviour

**Fig. 1:**
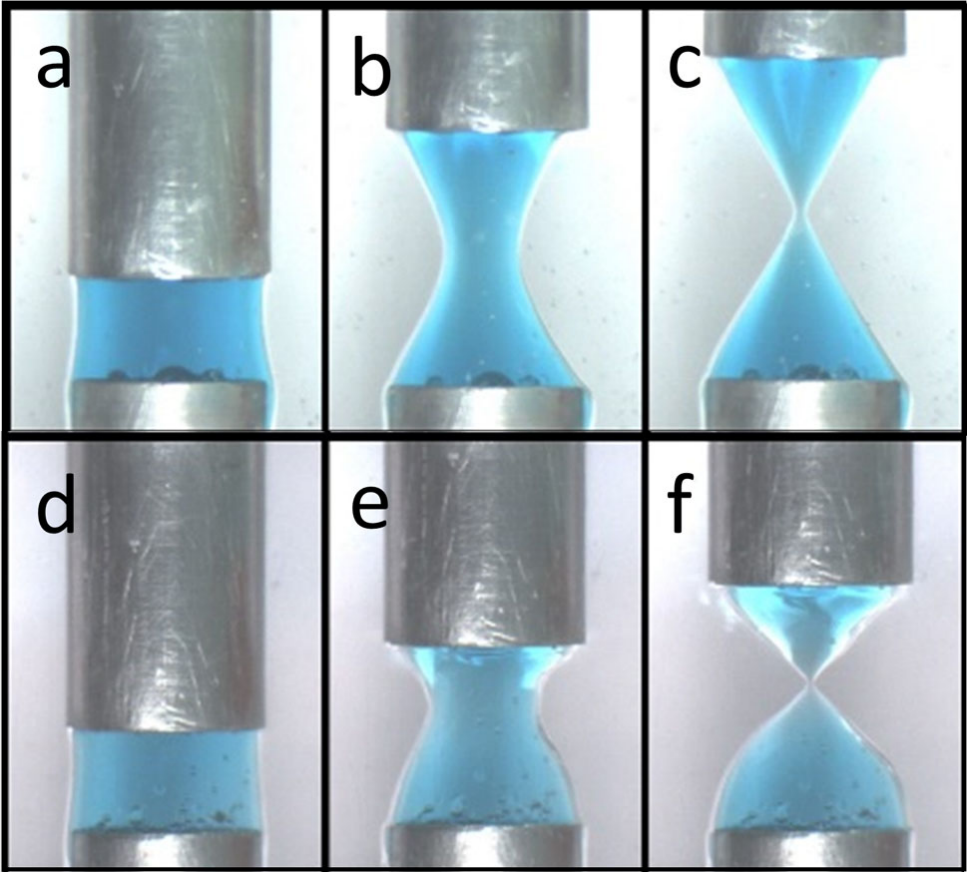
Extensional rheology of a 3wt% Laponite suspension having been left to age for **a**, **b**, **c** *t*_*w*_ = 0 or **d**, **e**, **f** 320 min following rejuvenation. Each row of pictures shows the initial fluid (**a**, **d**), the final shape just prior to break-up (**c**, **f**) and an intermediate point (**b**, **e**)

Figure 1 shows example images of a 3wt% Laponite suspension subjected to a constant extensional Hencky strain rate, $({\dot{H}}/H_{0}) = 0.08\,\hbox{s}^{-1}$. Images are shown of a starting configuration, an intermediate position and the sample just prior to break-up. The top row shows a sample which is measured immediately after the loading protocol (*t*_*w*_  ~ 0 min) and has thus been recently rejuvenated. The sample deforms in such a way that the sides are a close match to a hyperbola, finally resulting in a conical deposit. The second row of images in Fig. 1 shows a sample which was measured 320 min after the loading protocol (*t*_*w*_ = 320 min). Experiments conducted with samples of increased age have a profile that necks faster in the middle, resulting in an earlier break-up. As a corollary to this, it is visually apparent that the deformation accumulated in the region near to the end plates becomes negligible once significant necking begins.

In extensional rheology, for many types of complex fluid it is common to observe that, following initial necking, a thin cylindrical region develops between two masses of fluid adhered to each end plate [31]. This has been used in some studies to estimate either the yield stress or extensional viscosity given a knowledge of the interfacial tension [6, 15, 31]. Whilst our samples thin in the middle, we never observe such a filament. Rather the samples pinch off at relatively modest plate separations. To test whether the sample thinning is influenced by interfacial tension, we tried stopping the motion of the plates near to the final break-up point and then, waiting 60 s, before looking for changes in neck diameter. If none was observed, we moved the plates apart by an additional 25 µm and repeated until the two halves of the sample separated. Despite repeated attempts, it appeared that the final break-up behaviour was always induced by the small perturbation applied by moving the plates, rather than spontaneous thinning due to interfacial tension. However, this does confirm that the sample deformations in this study are due solely to the sample stress caused by motion of the rheometer plates.

### Characterising the aging of Laponite suspensions

It is clear in a qualitative fashion that aging of the Laponite samples effects their necking/break-up behaviour. Before returning to quantitatively characterise these dynamics in extension, we first perform shear measurements to understand how our sample’s rheological properties change with waiting time (*t*_*w*_). This is particularly important given the complicated stress history of the samples produced by injection.

At a fixed strain amplitude of 5%, for the 3 and 4wt% samples spanning the range of aging times considered, the storage modulus (G′) was found to be only weakly dependent on frequency over the range 0.02–20 Hz (see supplementary figure S2). G″ displays a slight minimum which is a common observation for soft glassy materials [32]. We therefore chose to perform all our oscillatory measurements at 1 Hz.

Strain amplitude sweeps at a frequency of 1 Hz showed that the linear-viscoelastic regime persists up to complex strains γ_lve_  ~  15%. This occurred at the same value for all concentrations studied and was not influenced by the aging of the samples. This observation matches those made in a recent study by Wendt et al. [33] who studied suspensions of another thixotropic clay, Bentonite. At strain amplitudes less than γ_lve_, G′ far exceeds G″ for all concentrations, with values of tan(*δ*) ranging from 0.05 for *t*_*w*_ = 0 to 0.02 for *t*_*w*_ = 320 min. Consequently, at low strains G′ ≫ G″ However, G′ remains significantly greater than G″ at higher strains (*γ*  ~ 50%), although the elasticity in this region is nonlinear and plastic deformations become increasingly important [34].

Choosing a single-strain amplitude of 5% (well within the linear visco-elastic regime) and a frequency of 1 Hz, we measured the growth in the elastic modulus with time since rejuvenation (*t*_*w*_). Measurements were made periodically at intervals of 10 min. The growth with time of the elastic modulus allows us to monitor the effect of aging on the sample. Despite only applying a small perturbation to the sample, we wanted to check that the specific measurement was not influencing the evolution of the sample microstructure. We therefore compared equivalent values (5% strain) obtained from strain amplitude sweeps performed for 3, 3.5 and 4wt% at 0, 45, 105 and 320 min. These latter measurements used a fresh sample each time, aged without any agitation, for each value of *t*_*w*_. These latter measurements agree well with the single measurements taken at 10-min intervals. This indicates that the measurement protocol did not have a significant influence on the structure of the Laponite suspension.

The measured values of G′ for each sample exhibited a two-stage power-law dependent growth. The exponents during both the initial and latter stages decrease with increase in sample concentration. A few studies of thixotropic clays (Bentonite [33], Na-Montmorillonite [14] and Laponite [14, 26]) have observed power-law growth in the measured value of G′ with waiting time. These studies, however, saw just a single exponent for each sample. However, all the concentrations studied here show a definite crossover between the initial growth in G′ and the later higher exponent. The crossover time decreases with sample concentration after some waiting time  ~  2.5–4 h. These exponents, together with the crossover times, are quantified for all samples in Table 1 in supplementary information.

To confirm that the crossover behaviour is not influenced by the specific measurement, we measured a 4wt% sample in the same way as before but with measurements taken at hourly rather than 10-min intervals (green diamonds). These measurements showed a crossover between the two exponents at a very similar aging time to the sample measured at 10-min intervals. The fact that our measurements were performed in oil also rules out possible effects due to evaporative losses.

**Fig. 2:**
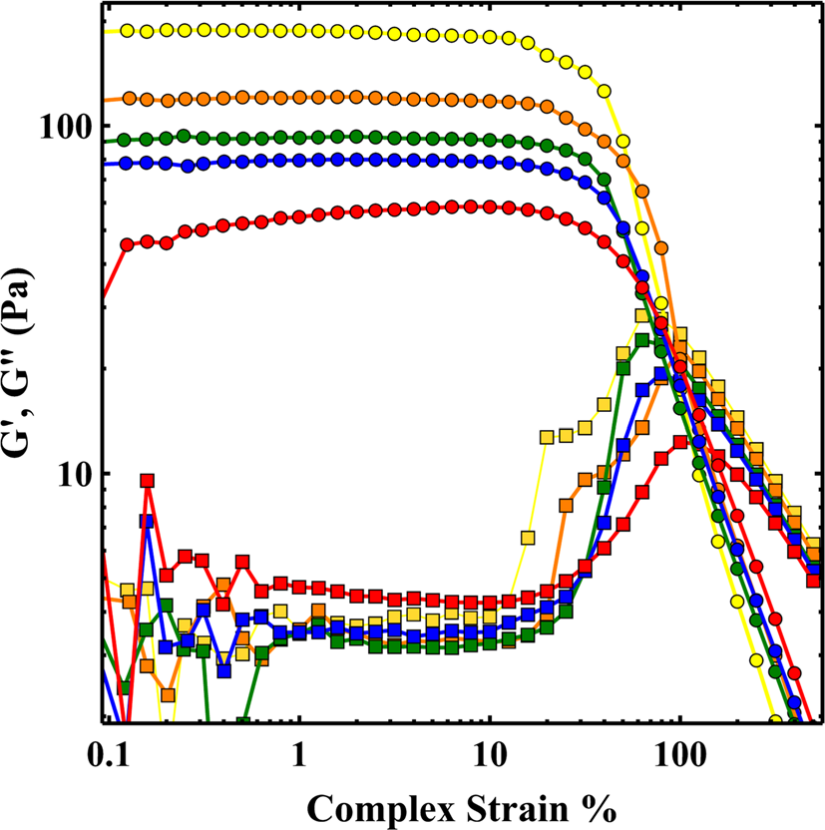
Strain amplitude sweeps at 1 Hz for a 3wt% sample at aging times of 0 (red), 45 (blue), 105 (green), 320 (orange) and 980 (yellow) minutes. Measurements of G′ (circles) and G″ (squares)

**Fig. 3:**
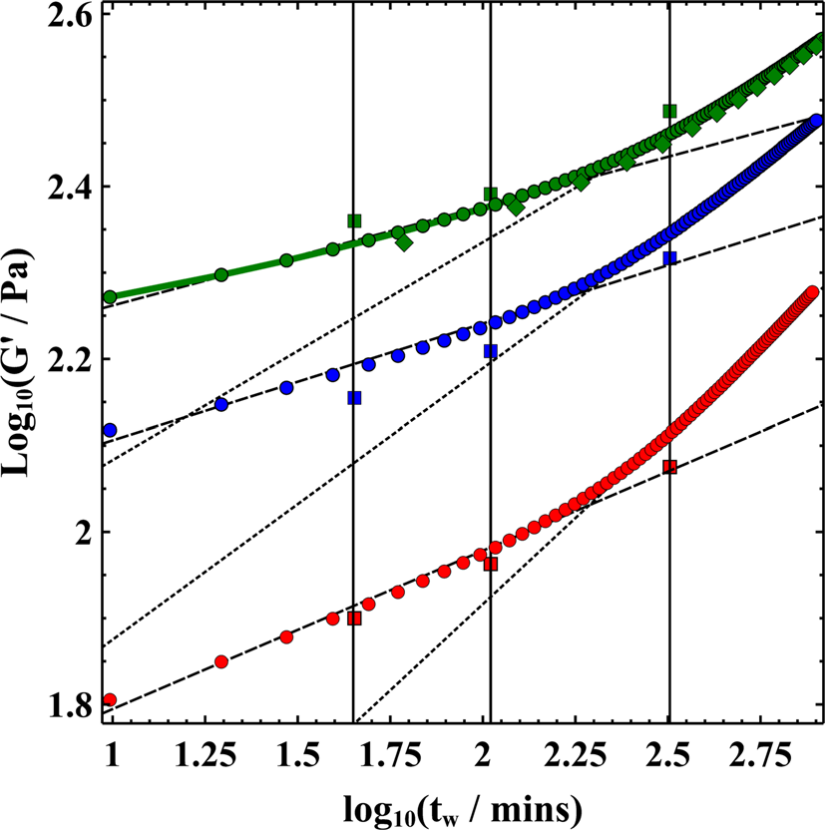
Aging of Laponite suspensions measured using oscillatory shear rheology. The growth in the elastic modulus G′ with waiting time is used to study the aging of Laponite suspensions at concentrations of 3wt% (red), 3.5wt% (blue) and 4wt% (green). Data is shown for several complementary methods, indicating that the measurements do not have a significant influence on the evolving microstructure. Samples were measured using a frequency of 1 Hz and a complex strain of 5% at periodic intervals of 10 min (circles) and 60 min (diamonds). The measured value of G′ at 5% strain was also plotted for samples subjected to a single-strain amplitude sweep at the indicated waiting time (squares). See Fig. 2 for example of a 3wt% sample. The growing elastic modulus exhibits a two-stage power law growth at all concentrations

A particularly intriguing feature of the strain amplitude sweeps in Fig. 2 is the appearance of a second peak or shoulder in the G″ measurements for samples at complex strains  ~  20%. This is absent in the freshly rejuvenated, *t*_*w*_ = 45, 105 min samples, but is seen very clearly in the *t*_*w*_ = 320 and 980 min datasets. The same transition in shape with aging time is seen in measurements of G″ for 3.5wt% and 4wt% samples. The appearance of this second peak fits well with the change in exponent observed in Fig. 3, suggesting the two phenomena may be linked.

Pham et al. [35] performed a comparison of repulsive hard-sphere glasses with those subject to attractive forces induced by depletion interactions using oscillatory shear rheology. They argued that whereas G″ for a repulsive glass exhibits a single peak due to cage breaking, attractive glasses have an additional peak at lower strains due to the breaking of inter-particle bonds. Whilst increases in gel stiffness occur due to the forming and rearrangement of the particles into more stable configurations within a glassy sample, it has also been shown in the context of silica gels that G′ can increase with *t*_*w*_ due to the stiffening of inter-particle bonds [36]. Under the conditions considered here (3–4 wt%, 10–4 M NaCl, pH 10), Laponite is believed to form a repulsive glass [17]. However, a 3wt% Laponite suspension (pH 10, 10^−4^ M NaCl) was observed to undergo a spontaneous transition from a repulsive Wigner glass to a DHOC glass with attractive interactions [19]. It should be noted that in that study this transition required aging times  ~ 3 days. However, as noted previously freshly prepared samples are known to age differently to those rejuvenated by shear stresses [19]. It would therefore be intriguing to find out if the change in exponents in Fig. 3 is due to the same type of transition, with timescales modified by rejuvenation. This should be possible with measurements of sample microstructure as performed in [19].

### Yield stress measurements and the growing stress overshoot

Measurements of the yield stress have a long and contentious history [37–39]. An important aspect of this debate has centred around the difficulty of measuring the yield stress [38, 40–42]. Different methods applied to the same fluids are known to result in different measured values of the yield stress [40]. The extensional measurements performed in this study are start-up rather than steady-state experiments in which relatively modest Hencky strains are applied. We therefore performed the most similar equivalent shear rheology experiment using a single constant shear-rate from start-up studying the build-up of visco-elastic stress in the sample, it’s deformation and yielding.

In the context of our extensional experiments, we are interested in how aging effects the initial yielding of the Laponite suspensions. In glassy thixotropic yield stress fluids, such as Laponite, particles in the quiescent fluid adopt progressively more energetically favourable local configurations with time. This can lead to a stress overshoot, such that the initial measured yield stress (static yield stress) differs from the value measured after the accumulation of significant strain (dynamic yield stress) [33]. Measurements of the static yield stress *σ*_ys_ have been defined either as the departure from linearity or the maximum measured stress [40, 42]. Here, we choose the maximum stress definition, but none of the conclusions below are significantly affected by such a choice. Our focus is on how the necking of samples scales with yield stress and yield strain.

**Fig. 4:**
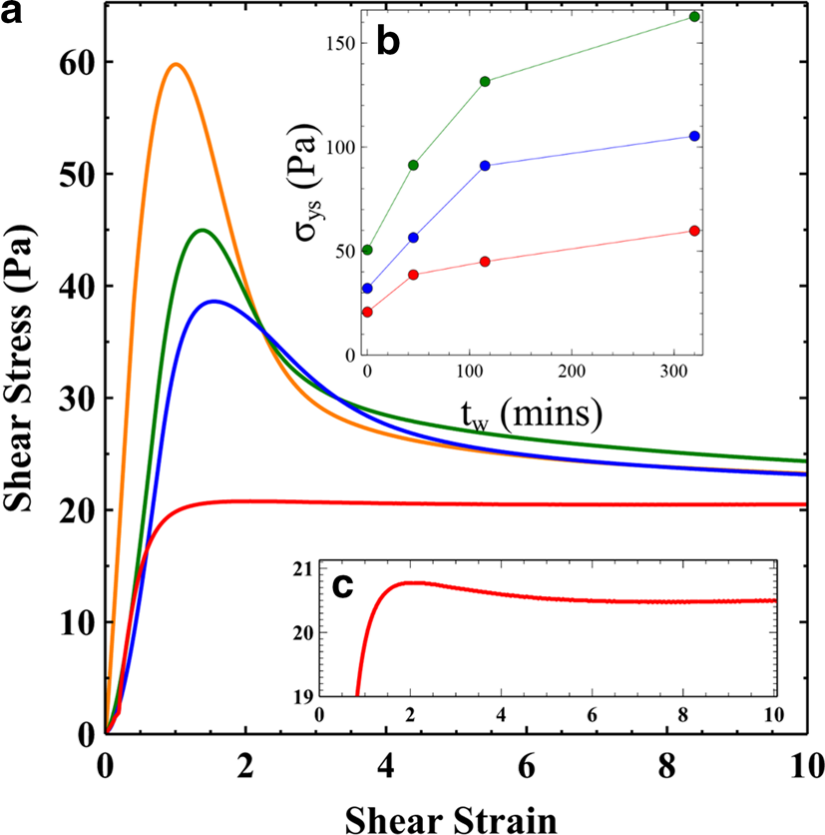
**a** shear stress v shear strain for 3wt% Laponite samples at aging times of 0 (red), 45 (blue), 105 (green) and 320 (orange) minutes. Samples show an increasingly large stress overshoot with a growing static yield stress (*σ*_ys_). The yield strain also reduces as the sample ages. **b** *σ*_ys_ as a function of sample age for Laponite concentrations of 3 (red), 3.5 (blue) & 4 wt% (green). c) 3wt%, *t*_*w*_ = 0 data showing that there still exists a very small stress overshoot

Figure 4a shows the shear stress–strain curves during a start-up experiment for 3wt% Laponite suspensions at different sample ages. The imposed shear rate was a constant value of 0.08 s^−1^, which was chosen to match the Hencky strain rate. Shear start up measurements were also performed for 3.5 and 4wt% samples at *t*_*w*_ = 0, 45, 105 and 320 min. These resulted in similar qualitative behaviour.

At *t*_*w*_ = 0 the stress–strain curves have a very slight overshoot (see Fig. 4c). However, as the samples age the overshoot becomes increasingly pronounced. Plots of *σ*_ys_ v *t*_*w*_ in Fig. 4b indicate that the sample aging is characterised by a growing static yield stress. The dynamic yield stress at large strains, however, tend to an approximately constant value. In addition to a growing yield stress, there is also a monotonic decrease in the yield strain, the strain at which the stress maximum is reached. Wendt et al. studying the thixotropic clay Bentonite in simple shear also observed an increase in the stress overshoot and a decreasing yield strain in samples allowed to age for longer [33]. They concluded that aging resulted in an increasingly brittle sample. This is also consistent with recent theoretical works on amorphous systems which argue that brittle failure is preceded by a strong stress overshoot which arises as the samples are allowed to age [11, 12].

### Thinning of the samples in extension

**Fig. 5:**
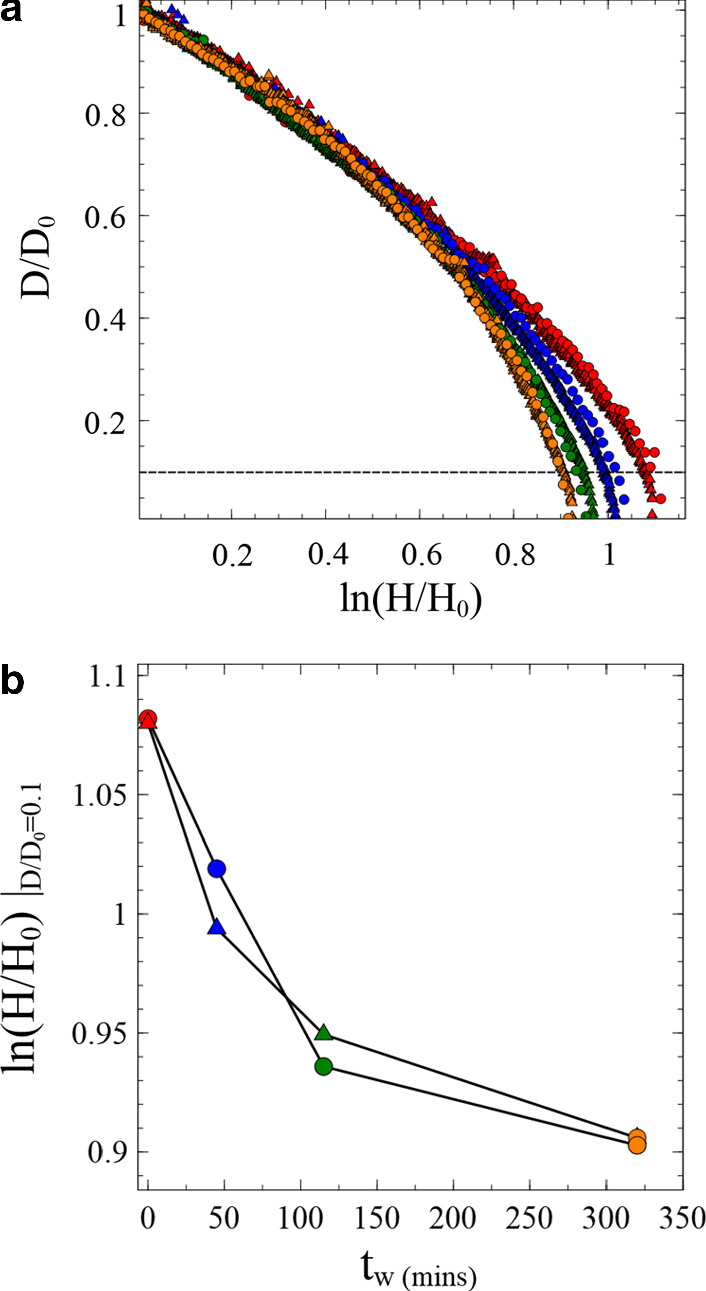
**a** The thinning of the midpoint of 3wt% Laponite suspensions after aging times of  ~  0 (red), 45 (blue), 115 (green), 320 (orange) mins. The samples initially follow the same curve up to a Hencky strain ln(H/H_0_)  ~ 0.6, where H_0_ and H represent the initial and current separation of the plates respectively. After this the more aged samples neck more rapidly. Data is shown for Hencky strain rates of 0.08 (circles) and 0.008 s^−1^(triangles). **b** The Hencky strain at a draw ratio D/D_0_ of the midpoint of the sample for different sample aging times, where D_0_ and D represent the initial and current diameter of the sample at its narrowest point. Colours and shapes are as above

To analyse the effect of aging on our extensional rheology experiments, we used image analysis software (see methods) to extract the diameter of the narrowest point in the fluid column, which was approximately at the midpoint. This was done for all collected images as the plates were moved apart. Figure 5a shows a series of measurements taken using the 3wt% Laponite suspension. The graph shows data for different aging times—0 (red), 45 (blue), 115 (green) and 320 (orange) minutes. There are also two types of experiments performed at different Hencky strain rates—0.08 s^−1^ (circles) and 0.008 s^−1^ (triangles). The experiments at different rates show very good agreement with one another. This illustrates two important points about the regime in which these experiments are being conducted. Firstly, although not surprising given our earlier characterisation of the aging of Laponite, this demonstrates that the timescale of experiments is not long enough for the aging of the Laponite during an experiment to have a significant impact. Simulations of aging amorphous fluids under an extensional geometry show that when the timescale of the experiment becomes comparable to the timescale of aging one should observe enhanced necking [29]. Secondly, the collapse of the datasets at these two rates shows that the experiments are quasi-static without strong flow-rate dependent viscous dissipation [30].

It is clear from Fig. 5a that all the samples initially follow the same flow curve up to a critical Hencky strain *ɛ*_*c*_  ~  0.6 regardless of sample age. At this point, the curves start to separate, which corresponds to the onset of necking, the rate of thinning as a function of plate separation from this point on does show a dependence on the age of the sample. The applied strain at this crossover point (*H*−*H*_0_)/*H*_0_  ~ 0.8 is comparable to the complex strain in oscillatory shear at which there is a strong increase in the value of G″ and decrease in G′ (i.e. just prior to the crossover of G′ and G″) indicating the accumulation of significant plastic strains at the onset of necking [43]. The significant increase in G″ appears to occur at strains that are independent of sample age. It is interesting that in our oscillatory rheology measurements the linear viscoelastic to nonlinear viscoelastic transition (the first point at which nonlinear elasticity and plastic effects can occur [44]) also shows no dependence on sample age. It also appears that our extensional measurements show the same behaviour with plastic deformation resulting in neck formation at the same extensional strain value, regardless of sample age. In contrast, the yield strains in our simple shear start-up measurements show a monotonic decrease with increasing sample age at all concentrations (Fig. 4). Whilst surprising, this same behaviour was observed by Wendt et al. in another thixotropic clay [33]. Collectively this supports a picture found in recent studies that prior to a sample yielding there are irreversible deformations that occur through significant accumulation of plastic strain. These become increasingly pronounced as the sample yield stress is approached [34].

At strains greater than the critical value ε_c_, the necking of more aged samples increases, resulting in elastoplastic flow in the necked region. This can be seen more clearly in Fig. 5b where we plot the Hencky strain at a draw ratio *D*/*D*_0_ = 0.1. This corresponds to Hencky strains  ~ 1 or (*H*−*H*_0_)/*H*_0_  ~ 1.7. Whilst a precise comparison between these values and the shear strains measured in Fig. 4 is not possible, it does indicate that the necking and thinning of the samples occurs at small strains in the vicinity of the initial yielding of the sample. The stresses generated in the samples by the moving plates, will therefore be strongly influenced by the sample’s aged structure. The quasi-static deformation of the sample will gradually begin to reorganise particles, but significantly greater strains would be required to fully rejuvenate the sample. Since the strain-rate-dependent term due to viscous losses is very small, the stress in the sample should be dominated by the yield stress [30]. At low strains, where rejuvenation is small, this should be close to the static yield stress of the sample.

**Fig. 6:**
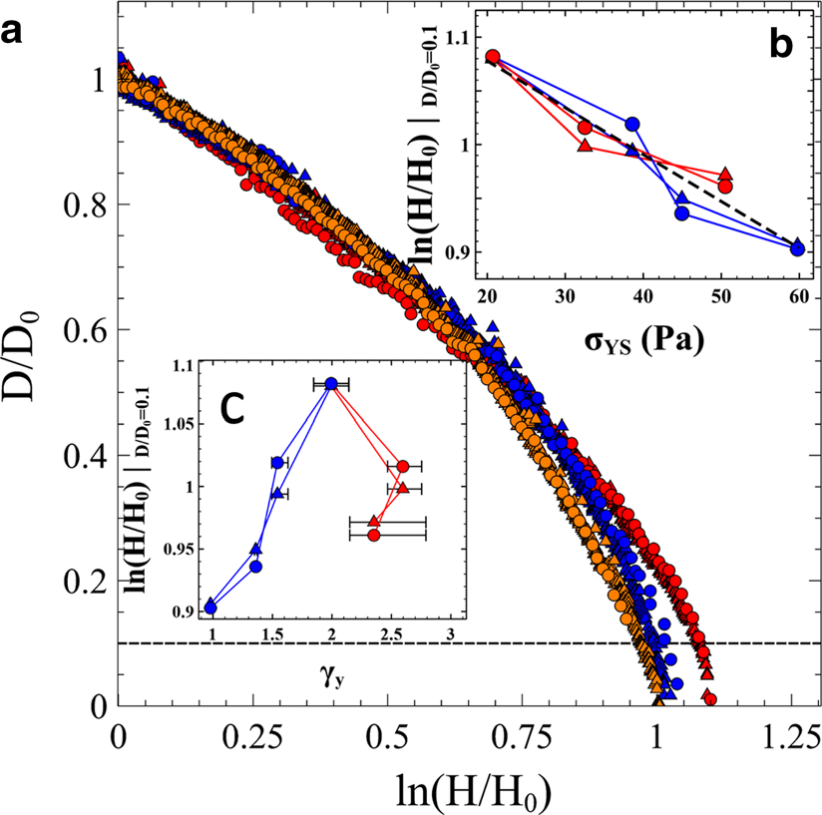
**a** Draw ratio D/D_0_ of extensional rheology experiments using 3 (red), 3.5 (blue) and 4wt% (orange). All samples were measured at an aging time *t*_*w*_  ~ 0 min. The curves show data collected at a Hencky strain rates of 0.08 (circles) and 0.008 s^−1^(triangles) illustrating that the degree of necking varies with the yield stress of the material. **b** The Hencky strain evaluated at a draw ratio of 0.1 (dotted line in main panel) as a function of sample yield stress. The data compares the 3wt% samples at aging times ranging from 0 to 320 min (blue) as shown in Fig. 5 with different sample concentrations shown in panel a (red). The degree of necking correlates well with the samples’ static yield stress. **c** In contrast the degree of necking does not indicate the samples’ yield strain

To understand more clearly the subsequent thinning behaviour of the different age samples, in Fig. 6 we compared the *t*_*w*_ = 0 samples for the 3, 3.5 and 4wt% samples. The shear–strain curves of all these samples exhibit a very small stress overshoot (see Fig. 4c) and although exhibiting significantly different yield stresses they yield at very similar values of the strain. The change in yield stress between the 3 and 4wt% samples, by chance, is also of comparable magnitude to the change in yield stress due to aging of the 3wt% sample for 320 min.

As the concentration of the Laponite is increased the samples neck faster. The flow curves separate at the same critical Hencky strain ɛ_c_ observed in our aging samples. To aid comparison between the data in Figs. 5a and 6a, we plot the value of the Hencky strain at a draw ratio *D*/*D*_0_ = 0.1 as a function of the corresponding yield stress (Fig. 6b) and yield strain (Fig. 6c) measured in simple shear. Red data is the different concentration data at *t*_*w*_ = 0 and the blue data is the 3wt% concentration samples at different values of *t*_*w*_. The circles represent data at a Hencky strain rate of 0.08 s^−1^ and the triangles represent data at 0.008 s^−1^.

An important factor in both sets of extensional measurements is the spatial distribution of strain within the samples. Indeed, the presence of necking indicates that the midpoint of the sample accumulates more plastic strain than the material near to the end plates. The stress at the midpoint also increases relative to the end plates since the constant axial force acts across different cross-sectional areas at different heights within the sample. Simulations of high yield stress fluids have shown that this can result in material yielding/flowing in the middle region but remaining unyielded at the end plates [45]. In this regime, where yield stress dominates effects due to interfacial tension, necking was shown to be more pronounced with increasing sample yield stress. Figure 6b indicates that in both sets of samples, at the slow imposed deformation rates, the thinning/necking behaviour correlates well with the sample yield stress [46]. Since the Hencky strains applied are still small, the aged samples show results consistent with the static yield stress rather than the dynamic yield stress.

**Fig. 7:**
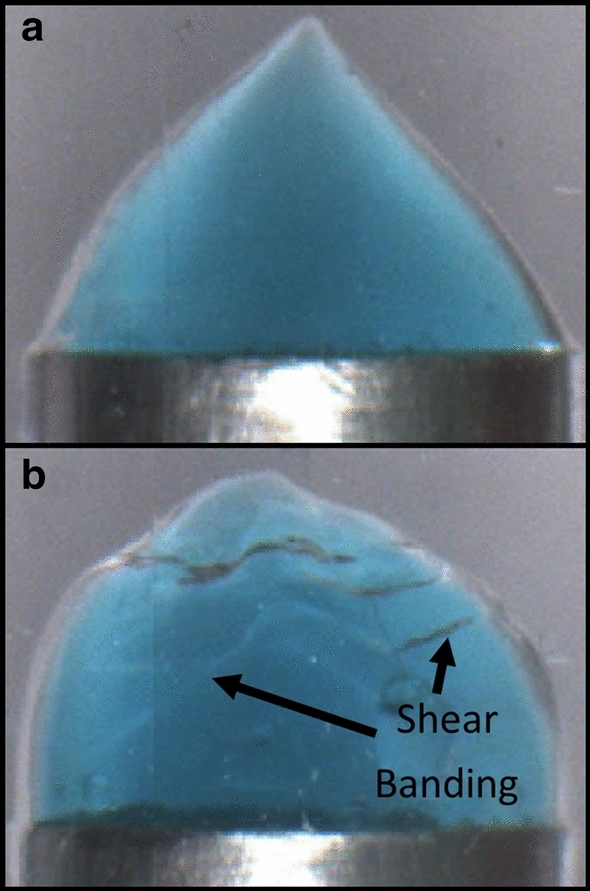
4wt% Laponite suspension after an experiment conducted at **a** *t*_*w*_ = 0 aging time and **b** *t*_*w*_ = 320 min. **a** Shows a ductile smooth break-up, whilst b shows features commonly associated with brittle break-up such as shear bands and possible fracture surfaces

However, it appears that the necking dynamics are not directly related to the sample yield strain (Fig. 6c). This is interesting in the context of discussions about assessing the relative ductile–brittle nature of soft materials when subjected to strain. More sudden break-up/faster necking is commonly interpreted as an indicator of a more brittle sample. Figure 7 shows the final images of 4wt% samples at aging times *t*_*w*_ = 0 and 320 min. In extension, increasing brittleness leads to a gradual transition from smooth flow, to shear banding to fracture [8–10]. Whilst our samples are generally ductile in nature, it is clear from visual inspection that the sample with greater age has developed shear bands indicating more brittle characteristics. It therefore seems correct to say that the Laponite suspensions become increasingly brittle as the samples age. This is consistent with the shear measurements, where reductions in a sample’s yield strain are also sometimes equated with a more brittle behaviour [33, 47]. One might therefore expect extensional necking/break-up as a function of sample Hencky strain to be a useful gauge by which a samples ductile–brittle nature could be compared. However, Fig. 6 shows that the curves only correlate with the sample yield stress and not the yield strain in shear. This despite the fact that the measurements cover similar changes in sample yield stress/elastic modulus. This highlights that whilst these extensional measurements can be used to compare the age of samples, the thinning profile is insufficient for an assessment of ductile-brittleness.

## Conclusions

We have shown that simple measurements of a Laponite suspension in an extensional geometry can be used to assess key features of the sample’s rheology. Following a critical extensional strain, the rate at which the sample’s neck decreases in diameter was shown to increase with a sample’s age. This increased necking is related to the fluids growing static yield stress but seems to be insensitive to changes in yield strain as determined in simple shear. These measurements suggest that following an initial linear visco-elastic regime, samples accumulate significant plastic deformations prior to the complete yielding of the sample. As samples age, they are also found to develop behaviour which is slightly more brittle, with older high concentration samples exhibiting shear bands.

## Supplementary Information

Below is the link to the electronic supplementary material.

Supplementary file1 (PDF 262 kb)

## Data Availability

The datasets generated during and/or analysed during the current study are available from the corresponding author on reasonable request.
