# Supplementary material for: Studying the aging of Laponite suspensions using extensional rheology
Source: Eur Phys J E Soft Matter. 2022 Nov 16;45(11):91. doi: 10.1140/epje/s10189-022-00244-9 (PMC9668775; doi:10.1140/epje/s10189-022-00244-9)
Supplement: Supplementary file 1 — Supplementary file1 (PDF 262 kb) [file 10189_2022_244_MOESM1_ESM.pdf]

Studying the aging of Laponite suspensions using extensional rheology – Supplementary Information

M.J. Hayes, M.I. Smith

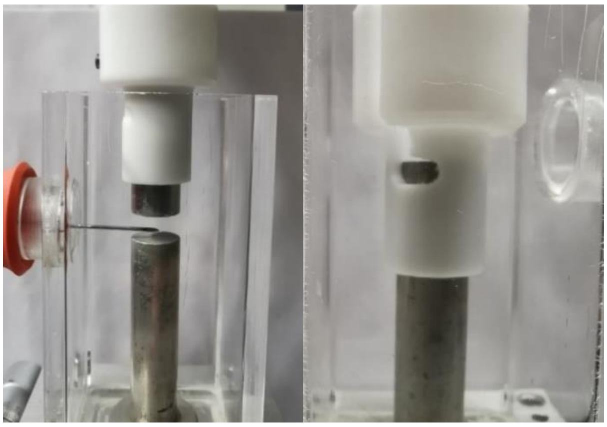

Figure S1: Photographs of the extensional setup. The lower plate is mounted inside a square cross-section Perspex tube which has a port, sealed with a rubber bung in the side wall. Laponite suspension is injected through this port over filling the gap between the two plates. In both images you can see the white PTFE collar used to trim the sample before measurements were taken. This collar is pushed down past the sample to remove the excess in one motion, generating a flat interface.

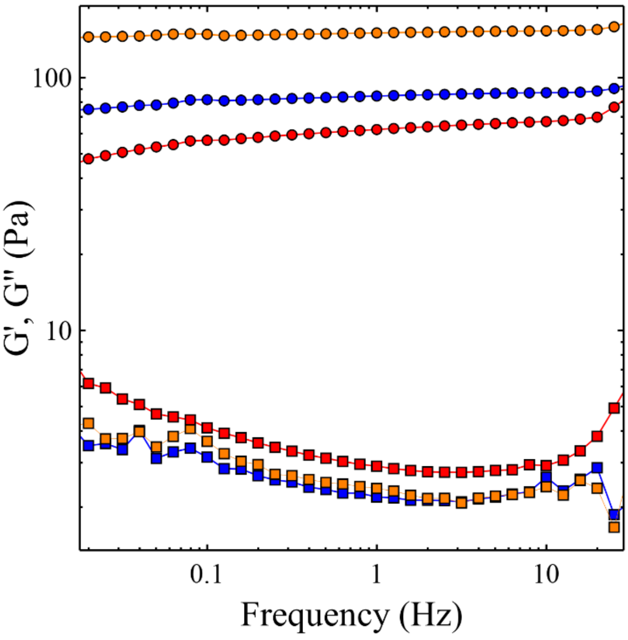

Figure S2: Oscillatory frequency sweeps at fixed strain amplitude of 5% on 3wt% Laponite samples aged for 0 (red), 45 (blue), 320 (orange) minutes. Data is shown for both G' the storage modulus (circles) and G" the loss modulus (squares).

Concentration (wt%)	First exponent	Second exponent	Crossover t_w (mins)
**3**	0.185	0.42	204
**3.5**	0.13	0.33	199
**4**	0.1	0.275	191

Table 1: Power law fits to G' aging data in figure 3
